# Feasibility and Acceptability RCT of a Piano Improvisation Intervention for Older Adults with and without MCI

**DOI:** 10.1002/alz70858_098662

**Published:** 2025-12-25

**Authors:** Julene K. Johnson, Jennifer Bugos, Theresa Allison, Jing Cheng, Karen Barrett, Charles Limb

**Affiliations:** ^1^ University of California San Francisco, San Francisco, CA, USA; ^2^ University of South Florida, Tampa, FL, USA; ^3^ University of California, San Francisco, San Francisco, CA, USA; ^4^ University of California San Francico, San Francisco, CA, USA

## Abstract

**Background:**

Older adults (age 65 and over) with mild cognitive impairment (MCI) are 3‐5 times more likely to progress to dementia than healthy counterparts. Engaging in cognitively challenging activities in later life is associated with decreased risk of cognitive decline. Music‐based training interventions are a promising strategy to address late‐life cognitive inactivity, but few interventions have been studied. The goal of this study was to conduct a pilot feasibility/acceptability RCT of a piano improvisation training to determine if a future mechanistic RCT was warranted.

**Method:**

We used RCT design with mixed‐methods data collection to examine the feasibility and acceptability of a recently adapted 12‐week, group piano improvisation intervention for older adults with and without MCI. We assessed standard feasibility and acceptability metrics. Participants were individually randomized and stratified by centers to either a small group (i) piano improvisation (experimental) or (ii) music listening (attention control) intervention. The interventions (with manuals) were led by a professional musician weekly for 12 weeks (90 minutes) with 4‐5 days of 30‐minute home practice. We administered pre‐ and post‐intervention assessments, anonymous satisfaction surveys, fidelity checks, and focus groups.

**Result:**

We assessed 70 individuals from three San Francisco senior centers for eligibility; 53 were enrolled (88% of enrollment goal) and randomized (mean age = 75.2 years, 77% female, and 69% non‐white). Thirty percent were classified as healthy and 70% as MCI (based on MoCA). Recruitment lasted 18 weeks (average 3/week), lower than our proposed 10/week (due to COVID). Study retention was 81% (exceeding the proposed 75%). Participants attended an average of 10/12 of the experimental and 6/12 of the control sessions. Missing survey data were rare. All participants in the experimental and 93% in the control group rated quality of the sessions as “good, very good, or excellent”. Focus group data supported survey findings.

**Conclusion:**

Our pilot RCT met a majority of our feasibility and acceptability thresholds, which supported the decision to move on to a larger, mechanistic trial. We are currently testing the hypothesis that piano improvisation training will improve self‐regulation among older adults, which in turn, promotes sustainment of cognitively challenging activities.

